# Risk attitude and belief updating: theory and experiment

**DOI:** 10.3389/fpsyg.2023.1281296

**Published:** 2023-12-21

**Authors:** Evelyn Y. H. Huang, Benson Tsz Kin Leung

**Affiliations:** ^1^Hong Kong Polytechnic University, Kowloon, Hong Kong SAR, China; ^2^Hong Kong Baptist University, Kowloon, Hong Kong SAR, China

**Keywords:** risk attitude, risk aversion, belief, learning, belief-based utility

## Abstract

Despite the importance of risk attitude in decision-making, its role in belief updating has been overlooked. Using economic theory, we analyzed a dual-self equilibrium where an individual first updates her belief about an uncertain state and then takes an action to maximize her payoff. We showed that stronger risk aversion drives more conservative actions and thus decreases the instrumental value of information relative to the importance of belief-based utility. As a result, the relationship between risk attitude and belief updating depends on the nature of the belief-based utility. With self-relevant information, stronger risk aversion leads to more belief change, whereas with self-irrelevant information, stronger risk aversion leads to less belief change. Our experimental results concur with the theoretical predictions with two settings where subjects update their belief about their IQ and a randomly drawn number, respectively. We discuss implications on persuasion, advertisements, and political campaigns.

## 1 Introduction

Research on risk attitude has received an abundance of attention across different disciplines including marketing, behavioral science, economic, and psychology (Weber et al., 2002; Wakebe et al., 2012). It affects individuals' financial decisions (Noussair et al., 2014; Oehler et al., 2018), career choices (Gaba and Kalra, 1999; Bonin et al., 2007; Jaeger et al., 2010; Argaw et al., 2017), medical decisions (Rosen et al., 2003; Arrieta et al., 2017; Massin et al., 2018), purchase and sales decisions (Okada, 2010; Shapiro, 2011; Jindal, 2015), etc. The existing research mainly focuses on the relationship between risk attitude and decision-making by assuming risk attitude is independent to belief updating, while there is scant knowledge about the relationship between risk attitude and belief updating.^1^ However, in many situations with information transmission, it is important to understand how people update their beliefs with new information in order to determine their subsequent decisions. For example, to evaluate the impact of information campaigns, e.g., campaigns to convey the importance of stay-home policy during COVID-19 (Krpan et al., 2021), it is crucial to understand whether information could effectively influence people's belief, and if yes, to what extent.^2^ This study aims to shed light on the role of risk aversion in belief updating and the underlying mechanism and discuss implications on persuasion, advertisements, and political campaigns.

From a Bayesian perspective, risk attitude has no impact on belief updating. Given a piece of information, and the understanding of the underlying information structure, individuals have no incentive to distort their belief as it will otherwise lead to sub-optimal decision-making in future.^3^ Given the popularity of the Bayesian paradigm, the literature has instead focused on how different characteristics of information structures affect belief updating. To give a few examples, Eil and Rao (2011) find evidence of asymmetric updating toward good and bad news in self-relevant but not self-irrelevant context, while Coutts (2019) found no evidence of asymmetric updating across different contexts; Alós-Ferrer and Garagnani (2023) found that larger incentive leads to a more reinforcing belief updating and less Bayesian updating; Coffman et al. (2023) showed that individuals are more likely to update to reinforce stereotypes.

In contrast, this study intends to investigate how risk attitude affects belief updating. Contributing to the research program of decision-making under uncertainty, our results suggest that there is an inherent relationship between risk preference and belief formation, which calls for more future research. It also sheds light on the mechanism behind the heterogeneous belief-updating behavior across individuals (see for example, Berlin and Dargnies, 2016; Sinclair et al., 2020), and could explain heterogeneous treatment effects in information provision experiments (Haaland et al., 2023). Moreover, it also has significant implications on persuasion, advertisement, politics, etc. First, belief updating behavior directly relates to consumers' susceptibility to being persuaded by advertisements. Our results hence speak to the empirical relationship between risk aversion and brand loyalty (Matzler et al., 2008) and between risk aversion and the effectiveness of advertisement (Jeong and Kwon, 2012). Second, our results also provide firms guidance on their advertisement strategy, depending on whether their target customers are more- or less- risk averse. Third, and similarly, our results also shed light on how politicians could target more- or less-risk-averse individuals more effectively in their political campaigns. Given the well-documented relationship between age and risk-aversion (Albert and Duffy, 2012), we also speak to the political divides between older and younger constituencies.

So, how would risk attitude affect belief updating? In this study, we first present an economic theory with the premise that individuals trade-off between the instrumental purpose and the non-instrumental (psychological) purposes of information. In a model of decision-making under uncertainty, the instrumental purpose of information refers to the need of improving decision-making: a more accurate belief enables the individual to better take into account available information and choose a better decision, e.g., to pick a better product or to vote for a better candidate. On the other hand, the non-instrumental purpose of information refers to the concept of belief-based utility such as motivated belief, a utility for reduced uncertainty, and updating cost (see Loewenstein and Molnar, 2018 for a review). We analyze a dual-self equilibrium where individuals first update their belief and afterwards take an action. Importantly, we show that individuals with stronger risk aversion choose more conservative actions and that diminishes the importance of the instrumental purpose of information relative to the non-instrumental purpose.^4^ As a result, more risk-averse individuals update their belief in a way that caters more to the non-instrumental purpose. In self-relevant settings, i.e., when the uncertainty is self-related, individuals have a higher demand for information (Bargh, 1982; Shapiro et al., 1997; Symons and Johnson, 1997; Gray et al., 2004; Sui et al., 2006; Turk et al., 2011), the non-instrumental purpose of information resembles the utility for reduced uncertainty, thus more risk-averse individuals update their belief more. On the other hand, in a self-irrelevant setting, i.e., when the uncertainty is not self-related, there is less utility for reduced uncertainty, updating cost becomes more (relatively) important; thus, individuals with stronger risk aversion update their belief less.

We then test our theoretical prediction in an experiment with two settings, where subjects have to update their belief with self-relevant and self-irrelevant information, respectively. We find that upon receiving the same information, subjects with stronger risk aversion update more in the self-relevant setting and less in the self-irrelevant setting. It, therefore, confirms our theoretical predictions. We also report a significant relationship between demographics, such as gender and confidence, and belief updating in both self-relevant and self-irrelevant settings. Combined with existing literature on gender differences, we argue that our findings on demographics and belief updating support our theory and the trade-off between instrumental and non-instrumental value of information.

This study is organized as follows. We present the theoretical analysis in the next section. Afterward, we present the experimental design and the results in Sections 3 and 4. Section 5 discusses potential concerns of our study. Lastly, we conclude by discussing the implications of our results.

## 2 Theory illustration

In this section, we present the theoretical foundation that illustrates how risk aversion affects belief updating. It sheds light on the mechanism behind the relationship between risk aversion and belief updating and helps formulate our hypotheses. In particular, we show that individuals with stronger risk aversion take more conservative actions and thereby have more incentive to form belief catering to non-instrumental objectives instead of instrumental objectives.

Imagine an individual who tries to learn an unknown state of the world to improve her decision-making. For example, she learns whether her IQ is among the top half of society in order to plan her future career or evaluates the quality of a social media platform to decide how much time she spends on it or predicts the state of the economy in the coming year for her investment plan. The state of the world is denoted as ω, and for simplicity, ω equals either 0 or 1. We assume that the two states are a priori equally likely.

**Figure 1:**
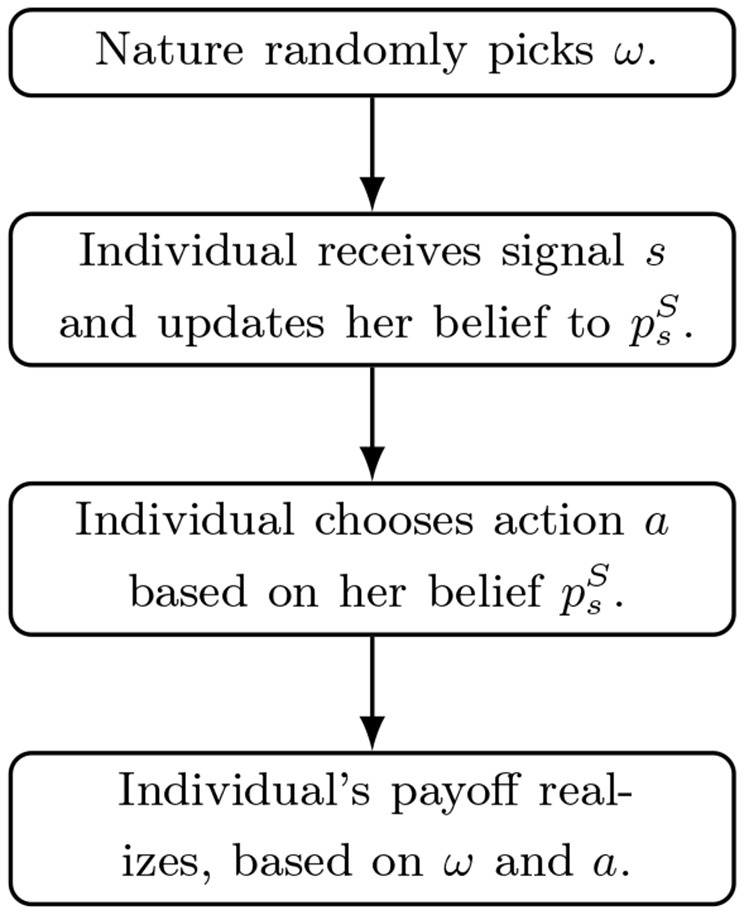
Timeline of the theory illustration.

In what follows, we analyze a scenario where the individual first receives a piece of information and updates her belief, and afterwards chooses her action based on her belief. The updating rule and action rule is characterized by a dual-self equilibrium introduced in the next paragraph. The timeline is illustrated in Figure 1. In period 0, nature randomly picks ω, which equals 0 or 1 with equal probability. In period 1, the individual receives a signal *s*∈*S*, which induces a Bayesian posterior in which we denote as $p_{s}^{B}$. For simplicity, we assume $p_{s}^{B}\sim U[\underline{p},\bar{p}]$, where $\bar{p}=1-\underline{p}$.^5^ Given the Bayesian belief $p_{s}^{B}$, the individual updates her belief to $p_{s}^{S}$ according to a linear updating rule $p_{s}^{S}=\left(1-\lambda \right)0.5+\lambda p_{s}^{B}$.^6^ In period 2, the individual chooses an action *a* according to a linear action rule $a=\left(1-\gamma \right)0.5+\gamma p_{s}^{S}$ and receives a payoff π^ω^(*a*) = 1−(ω−*a*)^2^ depending on the state of the world. To model risk aversion, we denote the utility function of the individual as *u*(π) = π^1−θ^ where θ∈(0, 1). A higher θ implies a stronger risk aversion.

The updating rule λ and the action rule γ are characterized as a dual-self Subgame Nash equilibrium, where the period-1 self first picks the updating rule λ and afterwards the period-2 self picks the action rule γ.^7^ The equilibrium solution is denoted as (λ^*^, γ^*^). Given our linear formulation, the period-2 self picks γ to maximize her expected utility denoted as *U*_2_:


$$U_{2}(a)=\int_{0.5+\lambda (\underline{p}-0.5)}^{0.5+\lambda (\bar{p}-0.5)}\left[p_{s}^{S}u(\pi^{1}(a))+(1-p_{s}^{S})u(\pi^{0}(a))\right]\frac{1}{\lambda (\bar{p}-\underline{p})}dp_{s}^{S}$$

On the other hand, for tractability of our analysis, we assume the period-1 self picks λ to solve the following minimization problem:


(1) $$\underset{\lambda }{\min}\int_{\underline{p}}^{\bar{p}}\left[{\left(a(p_{s}^{S})-a(p_{s}^{B})\right)}^{2}+V^{N}(p_{s}^{S})\right]\frac{1}{\left(\bar{p}-\underline{p}\right)}dp_{s}^{B}.$$

Equation (1) captures and allows us to focus on the main building block of the model, i.e., the trade-off between the instrumental and non-instrumental value of belief.^8^

The first item of Equation (1) corresponds to the instrumental value of belief, which is the quadratic difference between the Bayesian action and the action chosen by the period-2 self. The closer the period-2 self's action is to the Bayesian action, the lower of the first item is. It thus represents the utility loss of taking actions that is away from the Bayesian optimal action, i.e., the instrumental value of belief. The second item of Equation (1) corresponds to the non-instrumental value of belief, which we provide a few examples below.^9^

1. Motivated belief: for example, $V^{N}\left(p_{s}^{S}\right)=w\left(1-p_{s}^{S}\right)$. The individual gets higher utility if she believes state 1 is true.
2. Utility for reduced uncertainty: for example, $V^{N}\left(p_{s}^{S}\right)=-w{\left(p_{s}^{S}-0.5\right)}^{2}$. The individual gets higher utility if she is confident about the state.
3. Updating cost: for example, $V^{N}\left(p_{s}^{S}\right)=w{\left(p_{s}^{S}-0.5\right)}^{2}$. The individual incurs cost from updating her belief away from the prior.

Lastly, for ease of exposition, we assume that $\underline{p}$ is small enough such that *a* and $p_{s}^{S}$ are characterized by the first-order conditions and are inside [0, 1]. We proceed by backward induction and first characterize the action rule. The following proposition shows that individuals with stronger risk aversion are more conservative and choose action closer to 0.5.

**Proposition 1**. Denote the optimal action rule as $a^{*}=\left(1-\gamma^{*}\right)0.5+\gamma^{*}p_{s}^{S}$, γ^*^ decreases in θ.

The omitted proofs are shown in the Appendix. The intuition of Proposition 1 is as follows: as the degree of risk aversion θ increases, the individual has more incentive to insure herself against the mistake she would have made, or put differently, balance the utility between the two states. As a result, she does not tailor her action to her belief as much and chooses action closer to 0.5.

Now, we are ready to characterize the optimal belief updating rule $p_{s}^{S}=\left(1-\lambda^{*}\right)0.5+\lambda^{*}p_{s}^{B}$. Given our linear formulation and γ^*^, Equation (1) becomes


(2) $$\underset{\lambda }{\min}{\left(\gamma^{*}(1-\lambda )\right)}^{2}\text{Var}(p_{s}^{B})+\int_{\underline{p}}^{\bar{p}}V^{N}(p_{s}^{S})dp_{s}^{B}.$$

The first item of Equation (2) corresponds to the instrumental purposes of information. It is minimized at λ = 1 regardless of the value of γ^*^. Thus, if the second item of Equation (2) does not exist, the optimal belief updating rule is to update according to Baye's rule, which highlights the importance of belief-based utility (Loewenstein and Molnar, 2018). In contrast, in the presence of the non-instrumental purposes of information, the individual trades off between minimizing the two items in Equation (2). In particular, as shown in Equation (2), the instrumental value of information increases when $\text{Var}\left(p_{s}^{B}\right)$ increases, i.e., when the information is precise such that the Bayesian belief is more dispersed, or when γ^*^ increases, i.e., when the individual's action is more sensitive to his belief. The latter gives rise to our main theoretical result.

**Proposition 2**. A stronger risk aversion implies that individuals tailor their beliefs more to the non-instrumental than the instrumental purpose of information. For example,

1. if $V^{N}\left(p_{s}^{S}\right)=-w{\left(p_{s}^{S}-0.5\right)}^{2}$, $\frac{\partial \lambda^{*}}{\partial \theta }\geq 0$, i.e., individuals with stronger risk aversion updates more;
2. if $V^{N}\left(p_{s}^{S}\right)=w{\left(p_{s}^{S}-0.5\right)}^{2}$, $\frac{\partial \lambda^{*}}{\partial \theta }<0$, i.e., individuals with stronger risk aversion updates less.

Proposition 2 is driven by the result in Proposition 1. As individuals with stronger risk-aversion choose more conservative actions, i.e., as γ^*^ decreases, there is a lower cost of belief distortion, i.e., the first item of Equation (2) decreases. As a result, they have more incentive to update their belief catering to the non-instrumental purpose, i.e., the second item of Equation (2). In the first bullet point, the belief-based element of the loss function, i.e., $-w{\left(p_{s}^{S}-0.5\right)}^{2}$ decreases as $p_{s}^{S}$ is more extreme, thus representing the presence of utility for reduced uncertainty: utility loss decreases when the individual is more confident about the state. In such case, learning rate increases in risk aversion. In the second bullet point, the belief-based element of the loss function, i.e., $w{\left(p_{s}^{S}-0.5\right)}^{2}$ decreases as $p_{s}^{S}$ is closer to 0.5, thus representing the presence of an updating cost: utility loss increases when the individual's belief is more away from her prior belief. In such case, learning rate decreases in risk aversion.

Proposition 2 thus shows that the relationship between risk aversion and belief updating is context dependent. In the next section, we present our experimental result that tests our theory. We hypothesize our experimental results based on the two cases in Proposition 2.

## 3 Experimental design

We run the following experiment with two experimental conditions corresponding to the first and second bullet points of Proposition 2, which we call “SELF” and “NON-SELF” settings, respectively. The instruction could be found in the Online Appendix.

**Figure 2:**
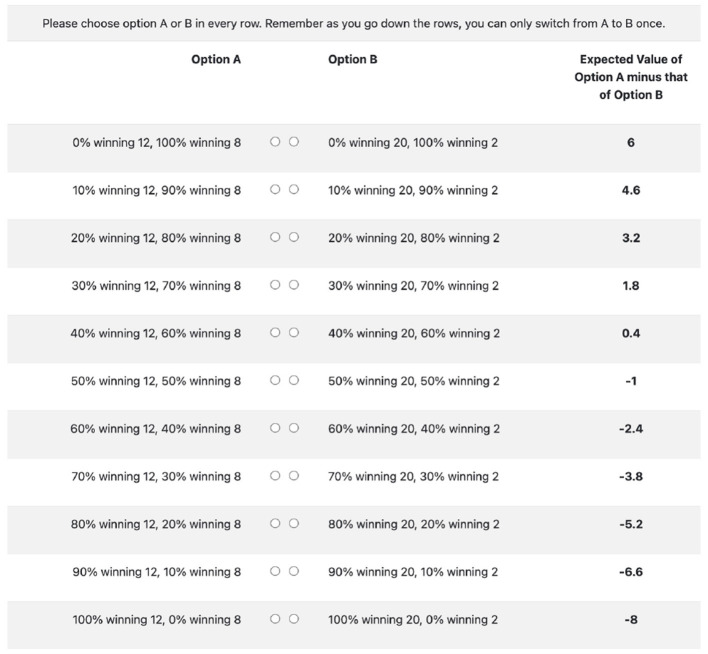
A multiple price list to elicit risk aversion. Subjects do not see the expected values. Note that a risk neutral individual should switch in the 6th row.

In both settings, subjects first fill out a demographic survey on their age, gender, and have to report their confidence about their own performance in a 20-question Raven Progressive Matrices test with a 5-point scale. Afterwards, we elicit the subjects' degree of risk aversion using a multiple-price list shown in Figure 2 (Holt and Laury, 2002). Option As are “safer” than option Bs. The subjects essentially decide on which row they switch from choosing option A to option B, the lower down they switch, the more risk averse they are.^10^ After that, subjects have to complete a 20-question Raven Progressive Matrices test within 20 min. Lastly, subjects have to guess and report their beliefs about a random variable that differs in the “SELF” and “NON-SELF” settings.^11^

**Figure 3:**
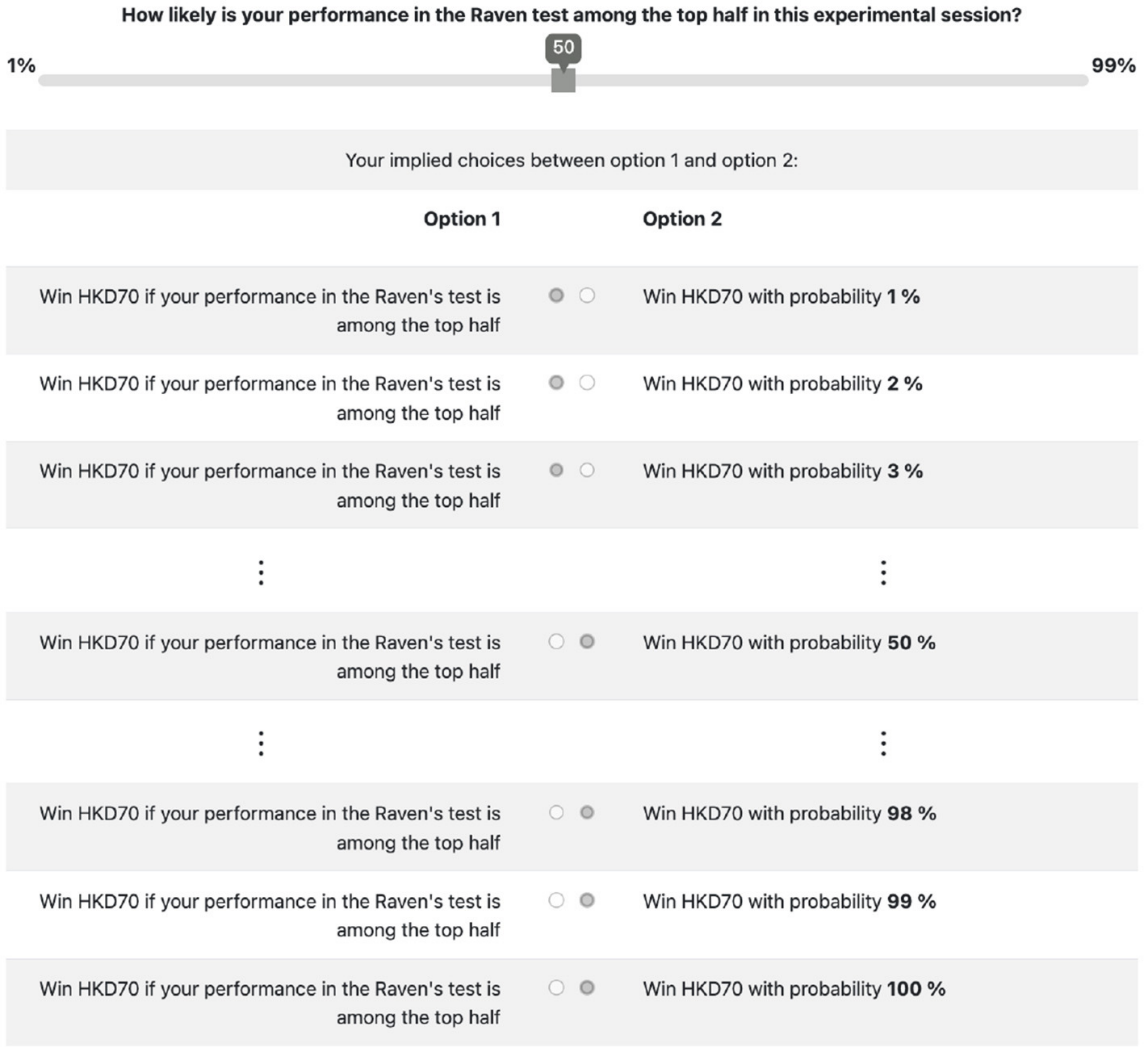
Table form of binarized scoring rule to elicit belief.

In the “SELF” condition, subjects have to form a belief about their performance in the Raven Progressive Matrices test. Therefore, the uncertainty is self-related.^12^ Without knowing their test results, they have to report their probabilistic belief that their result is among the top half of the session.^13^ We elicit their belief once right after the Raven test. We then provide them six pieces of information consecutively, and after each pieces of information, we elicit again their beliefs to track how they change. Thus, we elicit their belief seven times, which we denote as *p*_0_, *p*_1_, ⋯ , *p*_6_, using the table form of the binarized scoring rule as shown in Figure 3 (Hossain and Okui, 2013). Subjects have to indicate their beliefs using the slider, and the choices between option 1s and 2s are automatically selected which help to illustrate consequences of the binarized scoring rule. It is important to point out that risk preference does not affect belief elicitation using the binarized scoring rule.^14^ Between each elicitation, we provide them with a piece of information, which could be either a thumbs-up or a thumbs-down. If their result is among the top half, we show them a thumbs-up with a probability of 60%; if their result is among the bottom half, we show them a thumbs-down with a probability of 60%. The information structure is shown in Tables 1, 2 and is explained to the subjects.

**Table 1:** If subject's performance/random number is among the top half of the session.

**Generated signal**	** 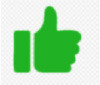 **	** 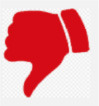 **
Probability of the signal	60%	40%

**Table 2:** If subject's performance/random number is among the bottom half of the session.

**Generated signal**	** 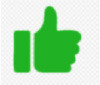 **	** 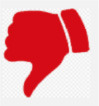 **
Probability of the signal	40%	60%

Next, we outline the “NON-SELF” condition. Rather than asking subjects to guess whether their performance in the cognitive ability test is among the top half or not, we ask subjects to guess whether a self-irrelevant, randomly drawn number is among the top half within the session. Formally, each subject is assigned a randomly drawn number from 1 to 100 with a uniform distribution, and the subjects are aware of this prior distribution. Similar to the “SELF” treatment, without telling the subjects their random number, we elicit subjects' probabilistic belief that their number is among the top half within the session seven times (once without information, and six times with information). The information, i.e., thumbs-up and thumbs-down, is generated by the same information structure in the “SELF” condition and is explained to the subjects. Note that the “NON-SELF” condition is “essentially equivalent” to the “SELF” treatment, except for the fact that the nature of the uncertainty is self-relevant in “SELF” and self-irrelevant in “NON-SELF.”

Given the extensive evidence, on the behavioral and neural level, that self-relevant information receives preferential attention (Bargh, 1982; Shapiro et al., 1997; Symons and Johnson, 1997; Gray et al., 2004; Sui et al., 2006; Turk et al., 2011), we hypothesize that the “SELF” setting resembles the utility for reduced uncertainty, i.e., the first bullet point of Proposition 2. Thus, subjects with stronger risk aversion update more in the “SELF” setting. Conversely, in the “NON-SELF” setting, as the utility for reduced uncertainty is absent (or at least reduced), updating cost becomes more (relatively) important.^15^ Thus, by the second bullet point of Proposition 2, subjects with stronger risk aversion update less in the “NON-SELF” setting.

## 4 Results

We have recruited 148 subjects via the university subject pool sign-UP system (Sona Systems; https://www.sona-systems.com). We run the “SELF” and “NON-SELF” sessions consecutively. In total, 74 subjects are in the “NON-SELF” and another 74 are in the “SELF” condition, giving us 148 × 6 = 888 data points of belief updating. The average age is 22.89, and 96 subjects are female. The summary of the demographics, along with other omitted statistical tests, can be found on the Online Appendix. We conducted the experiment in the behavioral laboratory at the university. Each session lasts around 1 h, and each subject earns 75 HKD. The experiment is approved by the Research Ethics Committee at Hong Kong Baptist University (REC/22-23/0023).

Our key variable of interest is the extent of belief updating of individuals, i.e., the “distance” between their prior and posterior beliefs, in which we quantify using the log-odds form of the Bayesian formula. With a prior belief *p*_0_ and upon receiving a thumbs-up, a Bayesian individual should update his belief to $p_{1}^{B}$ which follows:


$$\begin{array}{l} \log\frac{p_{1}^{B}}{1-p_{1}^{B}}=\log\frac{p_{0}}{1-p_{0}}+\log\frac{0.6}{0.4} \end{array}$$

where $\log\frac{0.6}{0.4}$ is the log-likelihood ratio of seeing a thumbs-up when the individual's performance/random number is among the top half versus when it is among the bottom half. Similarly, upon receiving a thumbs-down, a Bayesian individual should update his belief to $p_{1}^{B}$ which follows:


$$\begin{array}{l} \log\frac{p_{1}^{B}}{1-p_{1}^{B}}=\log\frac{p_{0}}{1-p_{0}}-\log\frac{0.6}{0.4}. \end{array}$$

Therefore, a Bayesian individual should update her belief by the magnitude of $\log\frac{0.6}{0.4}$ (upwards with good news and downwards with bad news). We denote this ratio ($\log\frac{0.6}{0.4}$) as *y*_Objective_ or log objective ratio. We denote the subjective analog of this log objective ratio by *y*_Subjective_ or log subjective ratio. With *p*_*i*_ denoted as the elicited belief after the *i*-th signal, and *p*_0_ denoted as the first elicited belief without any information, *y*_Subjective_ is defined as


$$y_{\text{Subjective}}=\{\begin{array}{cc} \log\frac{p_{i}}{1-p_{i}}-\log\frac{p_{i-1}}{1-p_{i-1}} & \text{upon receiving a thumbs-up}\\ \log\frac{p_{i-1}}{1-p_{i-1}}-\log\frac{p_{i}}{1-p_{i}} & \text{ upon receiving a thumbs-down} \end{array}$$

for *i* = 1, 2, 3, 4, 5, 6. *y*_Subjective_ thus measures how much the individual updates her belief upwards upon receiving a thumbs-up, and how much the individual updates her belief downwards upon receiving a thumbs-down. For a Bayesian individual, *y*_Subjective_ = *y*_Objective_.

### 4.1 Sanity check

We first check, using the data, whether subjects understand the information structure. More specifically, we regress the log subjective ratio with a regressor of log objective ratio^16^:


(3) $$\begin{array}{l} y_{\text{Subjective}}=\beta_{1}\times y_{\text{Objective}}+\epsilon . \end{array}$$

If the subjects do not understand the experiment and their belief updating process is totally random, β_1_ should be 0; if the subjects update their belief in the same direction as a Bayesian individual, β_1_ should be positive; if the subjects are perfectly Bayesian, both β_1_ and *R*^2^ should be equal to 1. The result is presented in Table 3.^17^ In both the “SELF” and “NON-SELF” condition, β_1_ is positive and significant. On average, subjects update upwards their belief upon receiving good news and downwards their belief upon receiving bad news. The subjects update their belief in the same direction suggested by Baye's formula, meaning that they understand the experiment setting and the information content of signals. Moreover, although both β_1_ in “SELF” and “NON-SELF” conditions are close to 1, the low *R*^2^ implies that there is significant heterogeneity across subjects on their belief-updating behavior. The significant heterogeneity is also shown in the box plot of log subjective ratio divided by log objective ratio in the Online Appendix.

**Table 3:** Regression analysis of log subjective ratio on log objective ratio (with standard errors in parentheses).

	**Dependent variable**	
	**Log subjective ratio**	
	**“SELF”**	**“NON-SELF”**
Log objective ratio	0.951^***^	1.009^***^
	(0.119)	(0.145)
Observations	444	444
*R* ^2^	0.125	0.099
Adjusted *R*^2^	0.123	0.097
Residual Std. Error (df = 443)	1.020	1.237
F Statistic (df = 1; 443)	63.442^***^	48.537^***^

^*^p < 0.1; ^**^p < 0.05; ^***^p < 0.01.

### 4.2 Risk attitude and belief updating

Next, for our main experimental result, we estimate the following regression^18^:


(4) $$\begin{array}{l} y_{\text{Subjective}}=\beta_{1}\times y_{\text{Objective}}+\beta_{2}\times \text{high risk aversion}\times y_{\text{Objective}}+\epsilon \end{array}$$

where “high risk aversion” is a dummy variable and is equal to 1 if the subjects' level of risk aversion is higher than the median.^19^ β_2_ thus measures the average difference between an subject with higher-than-median level risk aversion and an subject with lower-than-median risk aversion. We focus on the estimation of β_2_, where β_2_>0 implies that stronger risk aversion leads to more belief change and the subject's belief is more reactive to the received information, and β_2_ <0 implies that stronger risk aversion leads to less belief change. The result is presented in Table 4. Our estimation shows that β_2_ = 0.623 (*p* < 0.01) in the “SELF” condition, and β_2_ = −0.597 (*p* < 0.05) in the “NON-SELF” condition. The result thus provides evidence for our theoretical prediction, in which individuals with stronger risk aversion update more when the information is self-relevant, corresponding to a setting with utility for reduced uncertainty, and update less when the information is not self-relevant, where updating cost is more influential. The magnitude of the effect is also substantial: in the “SELF” condition, subjects who has high risk aversion updates almost twice ($\frac{0.623+0.648}{0.648}=1.96$ times) as much as the subjects who has low risk aversion; in the “NON-SELF” condition, subjects who has high risk aversion updates about half ($\frac{1.356-0.597}{1.356}=0.56$ times) as much as the subjects who has low risk aversion.

**Table 4:** Regression analysis on how risk aversion affect belief updating.

	**Dependent variable:**	
	**Log subjective ratio**	
	**“SELF”**	**“NON-SELF”**
Log objective ratio	0.648^***^	1.356^***^
	(0.165)	(0.223)
Log objective ratio × high risk aversion	0.623^***^	−0.597^**^
	(0.237)	(0.292)
Observations	444	444
*R* ^2^	0.139	0.107
Adjusted *R*^2^	0.135	0.103
Residual Std. Error (df = 442)	1.013	1.233
F Statistic (df = 2; 442)	35.588^***^	26.528^***^

^*^p < 0.1; ^**^p < 0.05; ^***^p < 0.01.

### 4.3 Demographics

**Figure 4:**
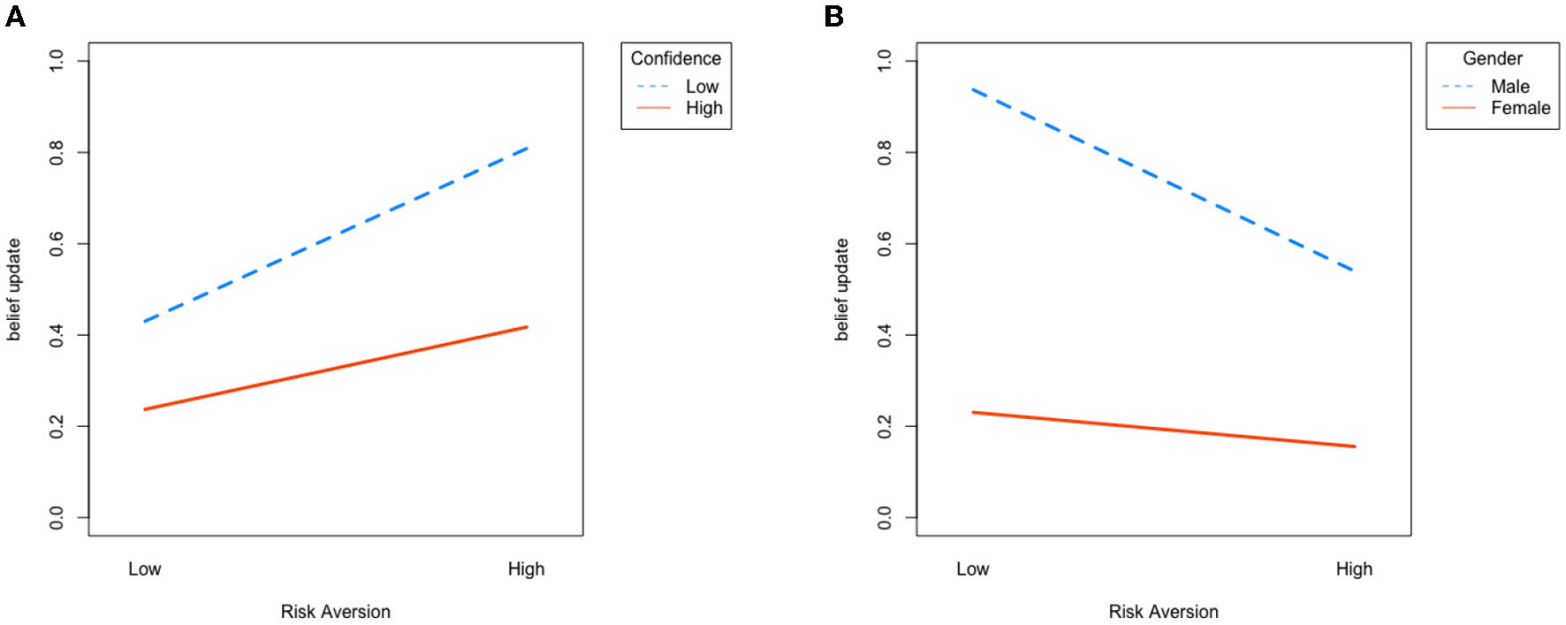
Interaction plot, where the y-axis is average log subjective ratio and the x-axis represents high and low risk aversion. **(A)** “SELF” condition: subjects with high risk aversion, or low confidence update more. **(B)** “NON-SELF” condition: subjects with high risk aversion, or who are female update less.

We also conduct regression analysis with demographic variables, including age, gender, and subjects' self-reported confidence in their Raven test. The result is shown in Table 5, and the interactive plot in Figure 4. First note that our main result remains significant: in the “SELF” condition, subjects with higher risk aversion update more (β_2_ = 0.634, *p* < 0.01); while in the “NON-SELF” condition, subjects with higher risk aversion update less (β_2_ = −0.543, *p* < 0.1).

**Table 5:** Regression analysis on how risk aversion affects belief updating, with demographic variables.

	**Dependent variable**	
	**Log subjective ratio**	
	**“SELF”**	**“NON-SELF”**
Log objective ratio	1.252	4.665^***^
	(1.018)	(1.114)
Gender	0.165	−0.592^***^
	(0.110)	(0.127)
Age	−0.006	−0.007
	(0.013)	(0.013)
Confidence	−0.122^**^	−0.080
	(0.054)	(0.062)
Log objective ratio × high risk aversion	0.634^***^	−0.543^*^
	(0.238)	(0.289)
Observations	444	444
*R* ^2^	0.157	0.150
Adjusted *R*^2^	0.147	0.141
Residual Std. Error (df = 439)	1.006	1.207
F Statistic (df = 5; 439)	16.318^***^	15.528^***^

^*^p < 0.1; ^**^p < 0.05; ^***^p < 0.01.

In the “SELF” condition, only confidence significantly affects belief updating. In other words, subjects who are more confident about their Raven score update less (coefficient = −0.122, *p* < 0.05). The result is verified with an ANOVA test [*F*_(1, 441)_ = 8.47, *p* = 0.0038]. By contrast, confidence does not play a role in the “NON-SELF” condition, but gender does affect belief updating. More specifically, males significantly update more than females in the “NON-SELF” setting (coefficient = −0.592, *p* < 0.01), where the result is supported by an ANOVA test [*F*_(1, 441)_ = 21.16, *p* < 0.01].

Note that both result on confidence and gender support our theory and the trade-off between the instrumental and non-instrumental value of information. Subjects with higher confidence have less demand of self-information, and less non-instrumental value, and thus update less with information. On the other hand, as males are more competitive than females (Croson and Gneezy, 2009; Buser et al., 2014; Saccardo et al., 2018), it suggests that males have a higher non-instrumental need of being precise in belief formation even when the information is self-irrelevant and therefore update more.

## 5 Potential concerns

In this section, we discuss potential concerns on our experimental setup and alternative explanations. The omitted tables of statistical tests can be found in the Online Appendix.

### 5.1 Risk aversion as a binary variable

Note that we use a binary variable to avoid making extra parametric assumptions, in particular on the linear relationship between risk aversion and belief updating. While we show in our theoretical model a monotonic relationship between risk aversion and belief updating, the model is silent on the precise parametric relationship, e.g., it depends on the functional form of *V*^*N*^, $\frac{\partial \gamma^{*}}{\partial \theta }$, etc. Assuming, for example, a linear relationship essentially makes our prediction extra sensitive to subjects extreme level of risk-seeking/risk-aversion comparing to subjects with moderate level of risk attitude, which is particularly problematic given that the majority (≈70%) of our subjects switch in the 6th, 7th, or 8th row in the risk-elicitation task.^20^ Given that most subjects' level of risk aversion is 6, 7, or 8, in an extension, we model the level of risk aversion as a 3-levels variable: 0 when subject switches in or before the sixth row, 2 when subject switches in or after the eigth row, and 1 otherwise. In the “SELF” condition, β_2_ = 0.23 (*p* = 0.013). In the “NON-SELF” condition, the result is less significant, i.e., β_2_ = −0.1854 (*p* = 0.104) but the direction remains consistent with our main result. In another extension, we exclude all subjects whose level of risk aversion is strictly lower than 6 or strictly higher than 8. Similarly, in the “SELF” condition, β_2_ = 0.4723 (*p* = 0.001). In the “NON-SELF” condition, β_2_ = −0.3591 (*p* = 0.147). Lastly, we use the level of risk aversion as a 12-levels variables as elicited. The result is less significant but the direction remains consistent with our main result. In the “SELF” condition, β_2_ = 0.107 (*p* = 0.23). In the “NON-SELF” condition, i.e., β_2_ = −0.05303 (*p* = 0.52). All extensions exclude subjects who always choose option A or option B, and the results are shown in the Online Appendix.

### 5.2 Overconfidence and motivated belief

In the “SELF” condition, we do find a better-than-average effect: subjects' average prior belief that they are in the top half of their experimental session is 61% (larger than 50%). However, we do not find evidence of motivated belief or asymmetric belief updating toward good and bad news (Coutts, 2019). First, the average last elicited belief (after six signals) is also roughly 61%, which is not larger than their prior belief (i.e., 61%). Our ANOVA analysis additionally shows that subjects do not significantly update more when they received good signals compared to bad signals [*F*_(1, 441)_ = 0.05, *p* = 0.8255]. The equal updating between good and bad signals rules out motivated belief and supports a utility for reduced uncertainty as mentioned in previous sections.

### 5.3 Risk preference elicitation

We are aware that the multiple price list in Figure 2 is an imbalance between risk-seeking and risk-averse preferences. This, however, does not affect our results. More specifically, we only require an ordinal elicitation of risk aversion: subjects who are more risk averse switch from option A to option B in the lower rows of the multiple price list but not a cardinal elicitation of risk aversion.

### 5.4 Decision errors

One potential confounding variable of our result is the correlation of decision errors between the risk elicitation and the belief formation task. However, we believe that it is highly unlikely, given the differences in results in the “SELF” and “NON-SELF” conditions. For example, if subjects who mistakenly report a higher risk aversion also mistakenly report a higher belief, it will induce a positive correlation between risk aversion and belief updating in *both* “SELF” and “NON-SELF” conditions. To explain the opposite results in the “SELF” and “NON-SELF” conditions, subjects who mistakenly report a higher risk aversion have to mistakenly report a higher belief in the “SELF” condition, but a lower belief in the “NON-SELF” condition, which we find highly unlikely.

## 6 General discussion and conclusion

In this study, we theoretically and experimentally show that higher risk aversion leads to a low instrumental need and a higher sensitivity to the non-instrumental need for information. With a psychological need for self-knowledge, i.e., in the “SELF” condition, where subjects receive self-relevant information about their IQ, stronger risk aversion leads to more belief updating. In contrast, when subjects receive self-irrelevant information such that updating cost is more influential, stronger risk aversion leads to less belief updating. Our experiment thus shows a context-dependent relationship between risk attitude and belief updating and also provides supportive evidence for the theory of belief-based utility (Loewenstein and Molnar, 2018).

Contributing to the research program of decision-making under certainty, our results suggest that risk preference and belief formation are inherently related, and thus, information intervention could have a heterogeneous impact on different individuals. The results speak to the practice and designs of future research on information provision experiments (Haaland et al., 2023). In particular, future research could benefit from collecting data on (elicited or self-reported) risk attitudes as it allows researchers to identify the heterogeneous treatment effects on individuals with different risk attitudes. In contrast, the absence of data on risk attitudes will likely mute the estimated treatment effect, as individuals with stronger (resp. weaker) risk aversion update their beliefs with self-irrelevant (resp. self-relevant) information to a lesser extent. Estimating such heterogeneous treatment effects is particularly important in health economics (e.g., Nyhan et al., 2014; Nyhan and Reifler, 2015) as the target audience includes vulnerable, elderly, citizens who are typically more risk averse.

Conceptually, this study complements previous research about risk aversion and information acquisition/avoidance (Mehrez, 1985; Willinger, 1989; Ho et al., 2021). For example, Ho et al. (2021) finds that more risk-averse participants choose to avoid information to avoid risks of acquiring unfavorable or inaccurate information. Our study supplements their findings as we analyze how individuals update their belief upon receiving information. Our findings therefore apply in many situations where information is involuntarily received, for example, via social media, advertisements, or political campaigns. Our results additionally offer a potential alternative explanation to the result in Ho et al. (2021): as individuals with stronger risk averse anticipate their over-reaction to self-relevant information, when information quality is unknown, they have more incentive to avoid information in advance to protect themselves from inaccurate or unfavorable information.

Lastly, our results have important implications on advertisement, communication, and persuasion, and on how to better persuade or convey information to risk-averse individuals. We expect our results to provide firms guidance for advertisement strategies as well as inspire future marketing research. For example, as the relationship between risk attitude and belief updating is context-dependent, our results suggest different framing of advertisement is needed to target more- or less-risk-averse consumers. In particular, relating information to oneself (more personally) compels more risk-averse individuals to learn more, while “context-neutral” information compels more risk-averse individuals to learn less. Thus, for firms that target risk-averse individuals, for example, insurance companies, a plain “facts and statistics” type of advertisement might not be as effective as advertisements that connect the product to the consumers on a personal level. More research needs to be done on how effective different advertisement works on different groups of consumers.

Similarly, our results have important implications for political campaigns. For example, to target older constituencies who are more risk averse (Albert and Duffy, 2012), politicians should have their messages framed with a higher self-relevance so as to emphasize the utility for reduced uncertainty, such as using metaphors in political campaigns (Musolff, 2017). Similarly, as older constituencies with stronger risk aversion update more in a self-relevant context, it potentially explains the increased polarization among elderly citizens (Boxell et al., 2017).

## Data availability statement

The raw data supporting the conclusions of this article will be made available by the authors, without undue reservation.

## Ethics statement

The studies involving humans were approved by Research Ethics Committee at Hong Kong Baptist University. The studies were conducted in accordance with the local legislation and institutional requirements. The participants provided their written informed consent to participate in this study.

## Author contributions

BL: Methodology, Project administration, Writing—original draft, Writing—review & editing. EH: Conceptualization, Methodology, Software, Formal analysis, Writing—original draft, Writing—review & editing.

## Footnotes

^1^Ho et al. (2021) looks into the relationship between risk preference and preference of information acquisition. In contrast, we study how individuals with different risk preferences update their belief upon receiving the same piece of information.

^2^See Haaland et al. (2023) for a literature review of information provision experiments. In the conclusion, we discuss the implications of our results in the literature.

^3^This is true even when there are uncertainties over the information structure, or quality of the information. More specifically, uncertainty over the information structure could be incorporated to the information structure itself, just like compound lotteries can be reduced to simple lotteries.

^4^As an extreme example, an infinitely risk averse individual always picks the safest action, thus information has no impact on her action, i.e., information has no instrumental value.

^5^The Bayesian posterior is given by

$$\begin{array}{l} p_{s}^{B}=\frac{\mathrm{Pr}\left(s|\omega =1\right)}{\mathrm{Pr}\left(s|\omega =1\right)+\mathrm{Pr}\left(s|\omega =0\right)}. \end{array}$$

Note that instead of specifying the signal distributions Pr(*s*∣ω = 1) and Pr(*s*∣ω = 0), we directly model the resulting Bayesian posterior as our primitive, akin to the approach in the information design literature (Bergemann and Morris, 2019).

^6^This linear formulation combined with the quadratic loss function introduced later brings tractability, and also imposes less cognitive demand on the individual (Compte and Postlewaite, 2019).

^7^See Bénabou and Tirole (2004), Brunnermeier and Parker (2005), and Wilson (2014) for examples of dual-self/multi-self models that study deviation from Bayesian updating.

^8^An alternative formulation is to assume that the period-1 self maximizes a sum of expected utility and a belief-based utility, i.e.,

$$\underset{\lambda }{\max}\int_{\underline{p}}^{\bar{p}}[\left[p_{s}^{B}u(\pi^{1}(a(p_{s}^{S})))+(1-p_{s}^{B})u(\pi^{0}(a(p_{s}^{S})))\right]-V^{N}(p_{s}^{S})]\frac{1}{\left(\bar{p}-\underline{p}\right)}dp_{s}^{B}.$$

This, however, introduces an arbitrary scaling effect: as θ increases, i.e., level of risk aversion increases, *u*^1−θ^ also changes (increases when *u* < 1 and decreases when *u*>1). Therefore, risk aversion affects the trade-off between instrumental and non-instrumental value in a way that arbitrarily depends on the magnitude of payoff and the functional form. Such scaling effect presents even in the extreme case where action does not depend on the belief. Equation (1) eliminates the scaling effect.

^9^The non-instrumental purpose of belief corresponds to the belief-based utility, as discussed in Loewenstein and Molnar (2018).

^10^Note that a rational individual should never choose option B in the first row and option A in the last row. Two of our 148 subjects chose option B in the first row, while six subjects chose option A on the last row. Our results do not change after excluding those eight subjects. The robustness test is presented in the Online Appendix.

^11^Although subjects do not have to pick an action as in the theoretical model, as the belief elicitation is incentivized, there is still an instrumental value of belief.

^12^See Eil and Rao (2011), Castagnetti and Schmacker (2022), and Oprea and Yuksel (2022) for similar setup.

^13^We break all ties randomly, and it is conveyed to the subjects.

^14^See Hossain and Okui (2013) for the mathematical proof and experiment. By contrast, with quadratic scoring rule, subjects with stronger risk aversion report beliefs closer to 0.5, as shown in both theoretical and experimental analysis (Erkal et al., 2020).

^15^We do not posit that updating cost is higher in the “NON-SELF” setting than in the “SELF” setting, but that the decrease in or absence of utility for reduced uncertainty implies that the updating cost affects individuals more in the “NON-SELF” setting than in the “SELF” setting.

^16^Note that there is no intercept term in the regression as $y_{\text{Objective}}=\log\frac{0.6}{0.4}$ for all data points.

^17^The regression tables in this study are generated using the Stargazer package in R (Hlavac and Hlavac, 2022).

^18^Note that we do not add “high risk aversion” as a separate predictor because of collinearity, as $y_{\text{Objective}}=\log\frac{0.6}{0.4}$ for all data points and “high risk aversion × *y*_Objective_” is perfectly correlated with high risk aversion. Note that *y*_Objective_ is constant because of our simple information structure illustrate in Tables 1, 2. We add the constant *y*_Objective_ in the regression for the ease of interpretation: β_1_ measures the extent to which subjects update their belief vis-a-vis a Bayesian, and β_2_ is the impact of risk attitude on β_1_.

^19^The median subject switches from option A to option B in the eighth row of the multiple price list shown in Figure 2. As a risk neutral individual should switch in the sixth row, our median subject is risk averse.

^20^Almost always fewer than 5 subjects switch in the other rows, in both the “SELF” and “NON-SELF” settings. The distributions are presented in the Online Appendix.

## Proofs

### A.1 Proof of proposition 1

Proof. The first derivative of *U*_2_ w.r.t. γ is

$$\begin{array}{l} \frac{\partial U_{2}(a)}{\partial \gamma }=\int^{\text{​}}[p_{s}^{S}u^{\prime }(\pi^{1}(a)\pi^{1^{\prime }}(a)\\ \;+(1-p_{s}^{S})u^{\prime }(\pi^{0}(a))\pi^{0^{\prime }}(a)]\frac{p_{s}^{S}-0.5}{\lambda (\bar{p}-\underline{p})}dp_{s}^{S}\\ \;=\int^{\text{​}}[p_{s}^{S}{\left(\pi^{1}\right)}^{-\theta }(2-2a)\\ \;-(1-p_{s}^{S}){\left(\pi^{0}\right)}^{-\theta }(2a)]\frac{p_{s}^{S}-0.5}{\lambda (\bar{p}-\underline{p})}dp_{s}^{S}. \end{array}$$

And the second derivative of *U*_2_ w.r.t. *a* is

$$\begin{array}{l} \frac{\partial^{2}U_{2}(a)}{\partial \gamma^{2}}=\int^{\text{​}}\frac{p_{s}^{S}-0.5}{\lambda (\bar{p}-\underline{p})}[p_{s}^{S}{\left(\pi^{1}\right)}^{-\theta }(-2)-\\ \;(1-p_{s}^{S}){\left(\pi^{0}\right)}^{-\theta }(2)-\theta p_{s}^{S}{\left(\pi^{1}\right)}^{-\theta -1}{\left(2-2a\right)}^{2}-\\ \;\theta (1-p_{s}^{S}){\left(\pi^{0}\right)}^{-\theta -1}{\left(2a\right)}^{2}]dp_{s}^{S}<0. \end{array}$$

Thus, γ^*^ is uniquely pinned down by the first order condition. Next, using implicit differentiation, we have

$$\begin{array}{l} \frac{\partial \gamma^{*}}{\partial \theta }=-\frac{\partial^{2}U_{2}(p_{s}^{S},a)}{\partial \gamma \partial \theta }/\frac{\partial^{2}U_{2}(p_{s}^{S},a)}{\partial \gamma^{2}}\\ \;=\int^{\text{​}}\frac{p_{s}^{S}-0.5}{\lambda (\bar{p}-\underline{p})}[p_{s}^{S}\log(\pi^{1}){\left(\pi^{1}\right)}^{-\theta }(2-2a)\\ \;-(1-p_{s}^{S})\log(\pi^{0}){\left(\pi^{0}\right)}^{-\theta }(2a)]dp_{s}^{S}/\frac{\partial^{2}U_{2}(a)}{\partial \gamma^{2}}\\ \;=\int^{\text{​}}\frac{p_{s}^{S}-0.5}{\lambda (\bar{p}-\underline{p})}p_{s}^{S}{\left(\pi^{1}\right)}^{-\theta }(2-2a)[\log(\pi^{1})\\ \;-\log(\pi^{0})]dp_{s}^{S}/\frac{\partial^{2}U_{2}(a)}{\partial \gamma^{2}} \end{array}$$

where the last equality is implied by the first order condition. When $p_{s}^{S}>0.5$, the first derivative $\frac{\partial U_{2}\left(a\right)}{\partial \gamma }{|}_{\gamma =0}>\;0$ and, thus, *a* > 0.5 and π^1^ > π^0^. Thus, $\frac{\partial \gamma^{*}}{\partial \theta }<\;0$ and the results follow.

### A.2 Proof of proposition 2

Proof. We first prove the first bullet point of the proposition. Equation (2) in the main text can be rewritten as

$$\begin{array}{l} \underset{\lambda }{\min}{\left(\gamma^{*}\left(1-\lambda \right)\right)}^{2}\text{Var}\left(p_{s}^{B}\right)-w\lambda^{2}\text{Var}\left(p_{s}^{B}\right) \end{array}$$

The first order condition is

$$\begin{array}{l} \begin{array}{l} -2\gamma^{*}(1-\lambda^{*})\text{Var}(p_{s}^{B})-2w\lambda^{*}\text{Var}(p_{s}^{B})=0\\ \;\Leftrightarrow \;\lambda^{*}=\frac{\gamma^{*}}{\gamma^{*}-w} \end{array} \end{array}$$

and the second-order derivative is $\left(2\gamma^{*}-2w\right)\text{Var}\left(p_{s}^{B}\right)$ which is positive if and only if *w* is small enough. When the second-order derivative is positive, as $\frac{\partial \gamma^{*}}{\partial \theta }<0$, the result follows. On the other hand, when the second-order derivative is negative, λ^*^ = ∞, and the result trivially follows.

Similarly, for the second bullet point of the proposition, Equation (2) in the main text can be rewritten as

$$\begin{array}{l} \underset{\lambda }{\min}{\left(\gamma^{*}\left(1-\lambda \right)\right)}^{2}\text{Var}\left(p_{s}^{B}\right)+w\lambda^{2}\text{Var}\left(p_{s}^{B}\right) \end{array}$$

The first order condition is

$$\begin{array}{l} -2\gamma^{*}(1-\lambda^{*})\text{Var}(p_{s}^{B})+2w\lambda^{*}\text{Var}(p_{s}^{B})=0\\ \;\Leftrightarrow \;\lambda^{*}=\frac{\gamma^{*}}{\gamma^{*}+w} \end{array}$$

and the second-order derivative is $\left(2\gamma^{*}+2w\right)\text{Var}\left(p_{s}^{B}\right)$ which is positive. Again, as $\frac{\partial \gamma^{*}}{\partial \theta }<0$, the result follows.
