# Supplementary material for: Risk attitude and belief updating: theory and experiment
Source: Front Psychol. 2023 Dec 21;14:1281296. doi: 10.3389/fpsyg.2023.1281296 (PMC10771331; doi:10.3389/fpsyg.2023.1281296)
Supplement: Supplementary file 1 [file Data_Sheet_1.PDF]

Online Appendix of "Risk Attitude and Belief Updating: Theory and Experiment"

November 27, 2023

1 Additional Tables and Graphs

Table 1: Summary of demographics in the "NON-SELF" condition. $N = 74$.

Statistic	Mean	St. Dev.	Min	Max
State (0: bottom half; 1: top half)	0.500	0.501	0	1
Raven Score	11.356	3.831	1	17
Confidence on Raven Score	3.203	1.014	1	5
Age	23.135	4.505	18	43
Math training (1: No Math training; 2: Had Math training)	1.311	0.463	1	2
Gender (1: male; 2: female)	1.581	0.494	1	2
Degree of Risk Aversion (Higher means more risk averse)	7.122	2.177	1	12
High Risk Aversion (0: Risk aversion < 7; 1: Risk aversion≥ 7)	0.581	0.494	0	1

Table 2: Summary of demographics in the "SELF" condition. $N = 74$.

Statistic	Mean	St. Dev.	Min	Max
State (0: bottom half; 1: top half)	0.514	0.500	0	1
Raven Score	12.500	3.171	3	18
First elicited belief	61.176	19.509	1	99
Last elicited belief	61.041	27.498	1	99
Confidence on Raven Score	3.162	0.902	1	5
Age	22.649	3.689	19	42
Math training (1: No Math training; 2: Had Math training)	1.203	0.402	1	2
Gender (1: male; 2: female)	1.730	0.445	1	2
Degree of Risk Aversion (Higher means more risk averse)	6.824	1.857	1	12
High Risk Aversion (0: Risk aversion < 7; 1: Risk aversion≥ 7)	0.486	0.500	0	1

Table 3: Distribution of levels of risk aversion.

	risk aversion	1	2	3	4	5	6	7	8	9	10	11	12
Number of subjects	"NON-SELF"	1	0	3	4	5	20	16	13	4	1	6	3
	"SELF"	1	0	1	2	7	24	16	12	5	1	1	3

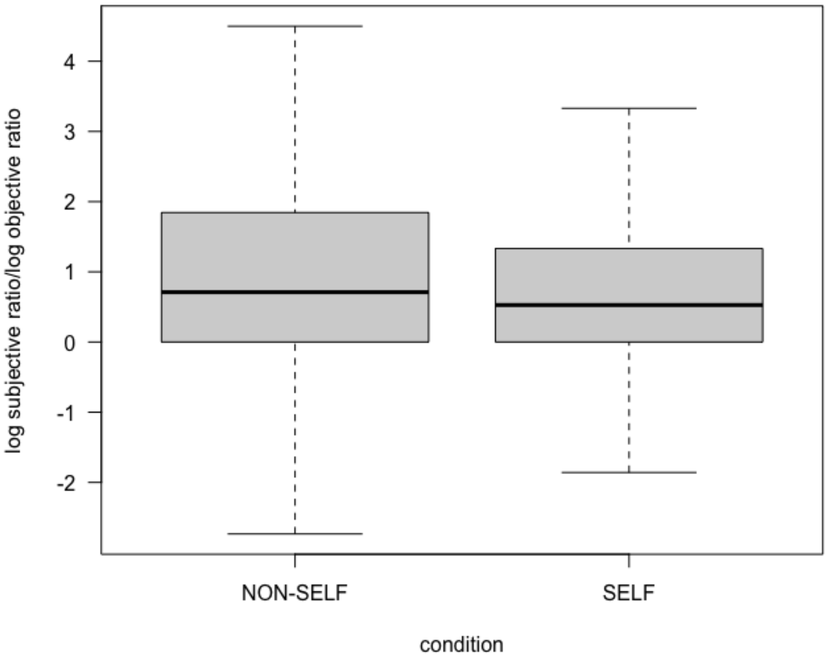

Figure 1: Box plot of log subjective ratio divided by log objective ratio, in "SELF" and "NON-SELF" condition. For illustrative purposes, we omit outliers in the box plot. A Bayesian individual's log subjective ratio should be equal to the log objective ratio. An individual updates to the same direction as an Bayesian individual if and only if their log subjective ratio divided by log objective ratio is positive. Therefore, while the majority of subjects update to the same direction as suggested by Baye's rule, we do see a significant amount of subjects who update in the wrong direction. Overall, we also see a significant heterogeneity across individuals' belief updating behavior.

Table 4: Regression analysis on how risk aversion affect belief updating, excluding subjects who always choose option A or always choose option B in the risk elicitation table.

	Dependent variable:	
	log subjective ratio	
	"SELF"	"NON-SELF"
log objective ratio	0.655***	1.407***
	(0.171)	(0.232)
log objective ratio×high risk aversion	0.571**	-0.677**
	(0.249)	(0.307)
Observations	420	420
R^2	0.127	0.107
Adjusted R^2	0.123	0.103
Residual Std. Error (df = 418)	1.031	1.262
F Statistic (df = 2; 418)	30.399***	24.983***
Note:	*p<0.1; **p<0.05; ***p<0.01	

Table 5: Regression analysis on how risk aversion affect belief updating, where risk aversion equals 0, 1, or 2: 0 when subject switches in or before the sixth row, 1 when subject switches in the seventh row, and 2 when subject switches in or after the eigth row. Subjects who always choose option A or option B in the risk elicitation task are excluded.

	Dependent variable:	
	log subjective ratio	
	(1)	(2)
log objective ratio	0.644***	1.295***
	(0.168)	(0.227)
log objective ratio x risk aversion	0.231**	-0.185
	(0.093)	(0.114)
Observations	420	420
R^2	0.129	0.102
Adjusted R^2	0.125	0.098
Residual Std. Error (df = 418)	1.030	1.265
F Statistic (df = 2; 418)	30.897***	23.760***
Note:	*p<0.1; **p<0.05; ***p<0.01	

Table 6: Regression analysis on how risk aversion affect belief updating, with only the subjects who switch in the 6th, 7th and 8th row.

	Dependent variable:	
	log subjective ratio	
	(1)	(2)
log objective ratio	-2.335**	3.476**
	(0.967)	(1.706)
log objective ratio x risk aversion	0.472***	-0.359
	(0.143)	(0.247)
Observations	318	294
R^2	0.167	0.087
Adjusted R^2	0.161	0.081
Residual Std. Error	0.837	1.388
F Statistic	31.585***	13.944***
Note:	*p<0.1; **p<0.05; ***p<0.01	

Table 7: Regression analysis on how risk aversion affect belief updating, where risk aversion ranges from 1 to 12 as elicited. Subjects who always choose option A or option B in the risk elicitation task are excluded.

	Dependent variable:	
	log subjective ratio	
	(1)	(2)
log objective ratio	0.207	1.391**
	(0.604)	(0.597)
log objective ratio x risk aversion	0.107	-0.053
	(0.088)	(0.082)
Observations	420	420
R^2	0.119	0.097
Adjusted R^2	0.115	0.093
Residual Std. Error (df = 418)	1.036	1.269
F Statistic (df = 2; 418)	28.246***	22.519***
Note:	*p<0.1; **p<0.05; ***p<0.01	

Table 8: ANOVA test in the "NON-SELF" condition, where the dependent variable is log subjective ratio. It shows that female updates significantly less than male.

	Coefficient
(Intercept)	1.36
gender (0: male; 1:female)	-0.52
log objective ratio × high risk aversion	-0.52

	Df	Sum Sq	Mean Sq	F value	Pr(>F)
gender	1	30.83	30.83	21.16	0.0000
log objective ratio×high risk aversion	1	4.88	4.88	3.35	0.0679
Residuals	441	642.48	1.46		

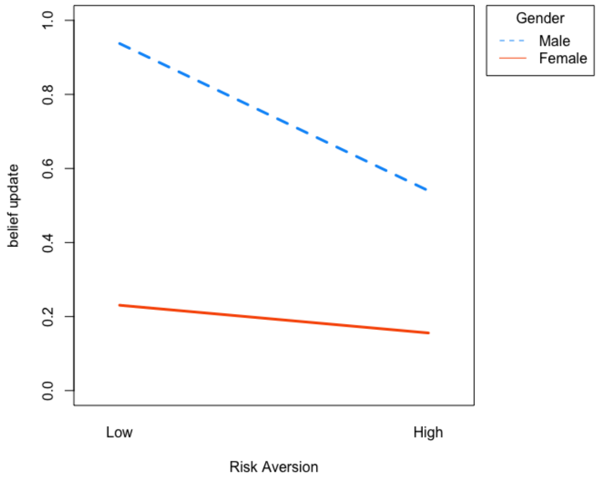

Figure 2: Interaction Plot of average log subjective ratio versus risk aversion, with male and female subjects in the "NON-SELF" condition. First, the downward slopes illustrate our main experimental result, that subjects with higher risk aversion update less in the "NON-SELF" condition. The fact that the blue dashed line is above the red solid line shows that male updates more than female in the "NON-SELF" condition.

Table 9: ANOVA test in the "SELF" condition, where the dependent variable is log subjective ratio. It shows that gender has no impact on belief updating.

	Df	Sum Sq	Mean Sq	F value	Pr(>F)
gender in experimental group	1	3.09	3.09	3.03	0.0824
log objective ratio×high risk aversion	1	7.86	7.86	7.71	0.0057
Residuals	441	449.75	1.02		

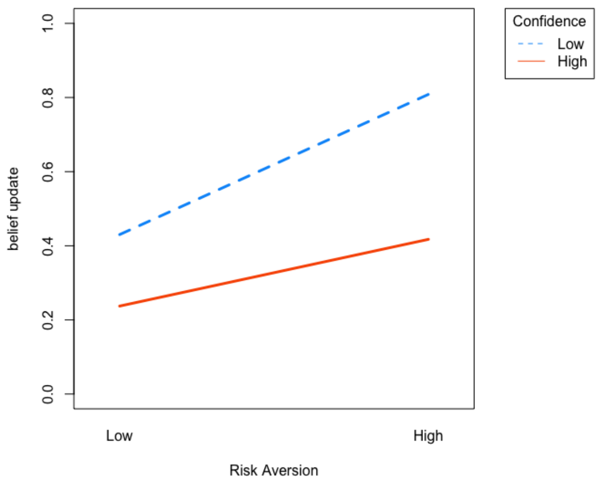

Figure 3: Interaction Plot of average log subjective ratio versus risk aversion, with subjects with high and low confidence in the "SELF" condition. We say that a subject has high confidence if and only if her reported confidence is bigger than the median. First, the upward slopes illustrate our main experimental result, that subjects with higher risk aversion update more in the "SELF" condition. The fact that the blue dashed line is above the red solid line shows that subjects with higher confidence update less in the "SELF" condition.

Table 10: ANOVA test in the "SELF" condition, where the dependent variable is log subjective ratio. "High confidence" is a dummy variable and is equal to 1 iff the subject is more confident than the median. It shows that confidence has a negative impact on belief updating.

	Coefficients
(Intercept)	0.75
high confidence	-0.31
log objective ratio×high risk aversion	-0.53

	Df	Sum Sq	Mean Sq	F value	Pr(>F)
high confidence	1	8.59	8.59	8.47	0.0038
log objective ratio×high risk aversion	1	5.03	5.03	4.96	0.0265
Residuals	441	447.08	1.01		

Table 11: ANOVA test in the "NON-SELF" condition, where the dependent variable is log subjective ratio. "High confidence" is a dummy variable and is equal to 1 iff the subject is more confident than the median. It shows that confidence has no impact on belief updating.

	Df	Sum Sq	Mean Sq	F value	Pr(>F)
high confidence	1	3.85	3.85	2.54	0.1118
log objective ratio×high risk aversion	1	6.01	6.01	3.97	0.0470
Residuals	441	668.32	1.52		

Table 12: ANOVA test in the "SELF" condition, where the dependent variable is log subjective ratio. It shows that the nature of signals (good or bad) has no impact on the magnitude of belief updating.

	Coefficients
(Intercept)	0.24
signals (0:bad signal; 1:good signal)	0.03
log objective ratio×high risk aversion	0.63

	Df	Sum Sq	Mean Sq	F value	Pr(>F)
signals (0:bad signal; 1:good signal)	1	0.05	0.05	0.05	0.8255
log objective ratio×high risk aversion	1	7.16	7.16	6.96	0.0086
Residuals	441	453.49	1.03		

Table 13: ANOVA test in the "NON-SELF" condition, where the dependent variable is log subjective ratio. It shows that the nature of signals (good or bad) has no impact on the magnitude of belief updating.

	Coefficients
(Intercept)	0.62
signals (0:bad signal; 1:good signal)	-0.14
log objective ratio×high risk aversion	-0.60

	Df	Sum Sq	Mean Sq	F value	Pr(>F)
signals (0:bad signal; 1:good signal)	1	2.08	2.08	1.37	0.2420
log objective ratio×high risk aversion	1	6.35	6.35	4.18	0.0414
Residuals	441	669.75	1.52		

2 Experimental Instructions

2.1 Instructions for the "SELF" condition

In this experiment, you will go through different cognitive tests. In this experiment, you can win from 50 to 140 HKD depending on your performance.

Stage 1: Survey Questions

In stage one you will have to answer some survey questions. Most of them are simple and should be clear. However, the last question deserve some attention. We will explain it in the following. The question is shown in the following figure:

Please choose option A or B in every row. Remember as you go down the rows, you can only switch from A to B once.

Option A		Option B	Expected Value of Option A minus that of Option B
0% winning 12, 100% winning 8	○ ○	0% winning 20, 100% winning 2	**6**
10% winning 12, 90% winning 8	○ ○	10% winning 20, 90% winning 2	**4.6**
20% winning 12, 80% winning 8	○ ○	20% winning 20, 80% winning 2	**3.2**
30% winning 12, 70% winning 8	○ ○	30% winning 20, 70% winning 2	**1.8**
40% winning 12, 60% winning 8	○ ○	40% winning 20, 60% winning 2	**0.4**
50% winning 12, 50% winning 8	○ ○	50% winning 20, 50% winning 2	**-1**
60% winning 12, 40% winning 8	○ ○	60% winning 20, 40% winning 2	**-2.4**
70% winning 12, 30% winning 8	○ ○	70% winning 20, 30% winning 2	**-3.8**
80% winning 12, 20% winning 8	○ ○	80% winning 20, 20% winning 2	**-5.2**
90% winning 12, 10% winning 8	○ ○	90% winning 20, 10% winning 2	**-6.6**
100% winning 12, 0% winning 8	○ ○	100% winning 20, 0% winning 2	**-8**

Note that there are 11 rows of A/B choices. In each row, you will have to pick one of option A and option B. As you can see, each option represents a lottery, and show two probabilities of rewards. For example, option A of the second row represents a lottery where you will win HKD 12 with probability 10% and HKD 8 with probability 90%, while option B of the second row represents a lottery where you will win HKD 20 with probability 10% and HKD 2 with probability 90%. In each row, you have to pick which choice is more attractive, and in the end of the experiment, we will randomly pick a row and pay you according to your choice.

Note that as you go down the rows, you can only switch from A to B once. Therefore, essentially you are choosing a "switching point". For example, you can choose to choose from option A to B in the forth row: it implies that you choose option A in the first, second and third row, and choose option B from the forth to the eleventh row.

Stage 2: Raven's Matrices

Stage 2 of the experiment involves a cognitive test which is widely used to measure IQ. It is called the Raven's Progressive Matrices. For each question, you have to decide which of option 1 to 8 is better fitted in the empty slot in the picture shown above the options. The figure below shows an example.

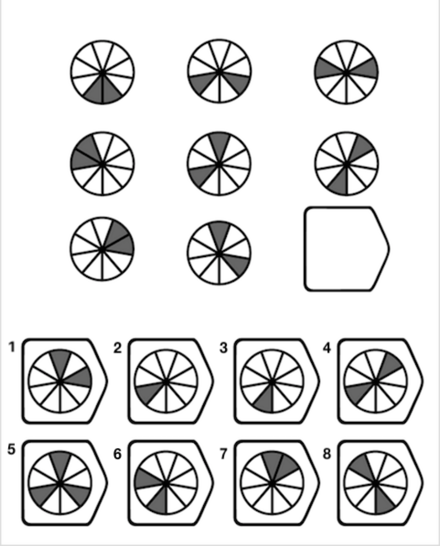

In stage 2, you will have to answer 20 questions in 20 minutes. The questions will be delivered to you as hard copy.

Stage 3: Guessing task

In stage 3, you have to guess whether your performance in the Raven's Progressive Matrices is among the top half or the bottom half of the sessions. You will have to indicate your guess seventh times (after seeing different information), using the following slider.

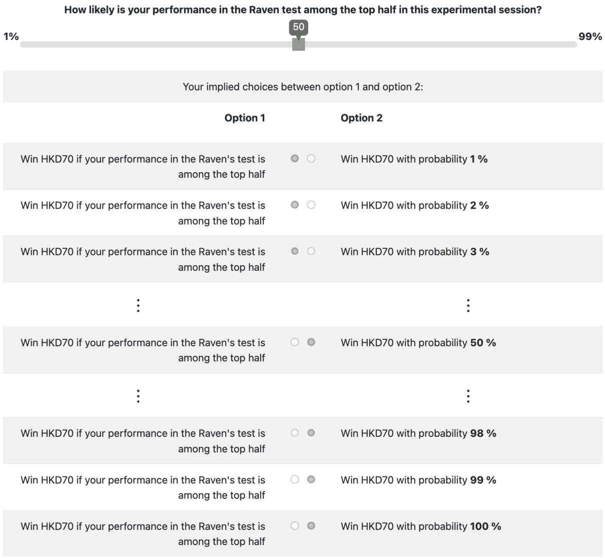

Figure 1: A slider to indicate your guess

As you can see in the Figure, you have to use the slider to indicate how likely you believe your performance is among the top half of the session, ranging from 1%to 99%. The table below the slider determines your experimental earning, and is designed in a way that you will be best off if you indicate your true belief.

The detail is as follows. There will be 100 different option 1/2 choices. In option 1, you will win HKD 70 if your performance in the Raven's test is among the top half. In option 2, you will win HKD 70 with a probability ranging from 1% to 99%. So if you believe that with 70% probability that your performance is among the top half, you should choose option 1 for the first 69 rows, and choose option 2 for row 70 to 100. Your choice in the slider will automatically determine your choices in the table. And therefore it is of your best interest to indicate your true belief. At the end, we will randomly draw one row from one of the seven guesses and pay you according to your choice.

As mentioned above, you will have to indicate your guess seventh times. First, you have to indicate your guess on how likely your performance is among the top half right after the Raven's test. Next, we will give you some information each time you indicate your guess.

More specifically, we will show you a thumbs-up or a thumbs-down depending on your performance. Figure 1 and 2 show the details. If your performance is among the top half, we will more likely show you a thumbs-up (with probability 60%); if your performance is among the bottom half, we will more likely show you a thumbs-down (with probability 60%). Therefore a thumbs-up or a thumbs-down gives your a hint about your performance, but they are not conclusive. It is possible that you still receive a thumbs-down even if your performance is among the top half, and receive a thumbs-up even if your performance is among the bottom half. You will receive six signals, and indicate six guesses along with it. For example, you see a thumbs-up, indicate a guess, then see another thumbs-up, revise your guess, then see a thumbs-down, revise your guess, etc.

generated signal	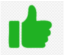	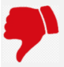
probability of the signal	60%	40%

Table 1: If you Raven's performance is among the top half of the session.

generated signal	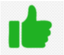	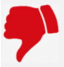
probability of the signal	40%	60%

Table 2: If you Raven's performance is among the bottom half of the session.

2.2 Instructions for the “NON-SELF” condition

In this experiment, you will go through different cognitive tests. In this experiment, you can win from 50 to 140 HKD depending on your performance.

Stage 1: Survey Questions

In stage one you will have to answer some survey questions. Most of them are simple and should be clear. However, the last question deserve some attention. We will explain it in the following. The question is shown in the following figure:

Please choose option A or B in every row. Remember as you go down the rows, you can only switch from A to B once.

Option A		Option B	Expected Value of Option A minus that of Option B
0% winning 12, 100% winning 8	○ ○	0% winning 20, 100% winning 2	**6**
10% winning 12, 90% winning 8	○ ○	10% winning 20, 90% winning 2	**4.6**
20% winning 12, 80% winning 8	○ ○	20% winning 20, 80% winning 2	**3.2**
30% winning 12, 70% winning 8	○ ○	30% winning 20, 70% winning 2	**1.8**
40% winning 12, 60% winning 8	○ ○	40% winning 20, 60% winning 2	**0.4**
50% winning 12, 50% winning 8	○ ○	50% winning 20, 50% winning 2	**-1**
60% winning 12, 40% winning 8	○ ○	60% winning 20, 40% winning 2	**-2.4**
70% winning 12, 30% winning 8	○ ○	70% winning 20, 30% winning 2	**-3.8**
80% winning 12, 20% winning 8	○ ○	80% winning 20, 20% winning 2	**-5.2**
90% winning 12, 10% winning 8	○ ○	90% winning 20, 10% winning 2	**-6.6**
100% winning 12, 0% winning 8	○ ○	100% winning 20, 0% winning 2	**-8**

Note that there are 11 rows of A/B choices. In each row, you will have to pick one of option A and option B. As you can see, each option represents a lottery, and show two probabilities of rewards. For example, option A of the second row represents a lottery where you will win HKD 12 with probability 10% and HKD 8 with probability 90%, while option B of the second row represents a lottery where you will win HKD 20 with probability 10% and HKD 2 with probability 90%. In each row, you have to pick which choice is more attractive, and in the end of the experiment, we will randomly pick a row and pay you according to your choice.

Note that as you go down the rows, you can only switch from A to B once. Therefore, essentially you are choosing a “switching point". For example, you can choose to choose from option A to B in the forth row: it implies that you choose option A in the first, second and third row, and choose option B from the forth to the eleventh row.

Stage 2: Raven's Matrices

Stage 2 of the experiment involves a cognitive test which is widely used to measure IQ. It is called the Raven's Progressive Matrices. For each question, you have to decide which of option 1 to 8 is better fitted in the empty slot in the picture shown above the options. The above figure shows an example. In stage 2, you will have to answer 20 questions in 20 minutes. The questions will be delivered to

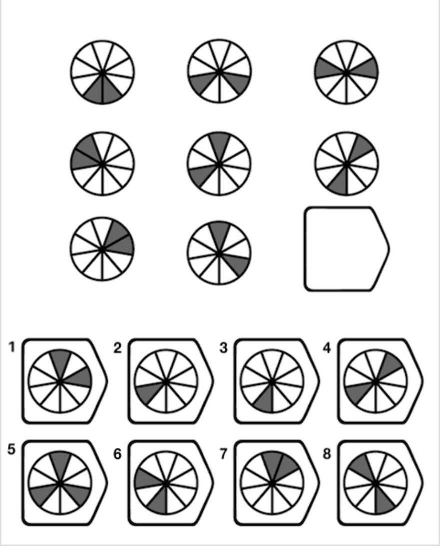

you as hard copy.

Stage 3: Guessing task

In stage 3, we will randomly and secretly draw a number from 1 to 100 for each of you. And you have to guess whether your number is among the top half or the bottom half of the sessions. You will have to indicate your guess seventh times (after seeing different information), using the following slider shown in Figure 1.

As you can see in the Figure, you have to use the slider to indicate how likely you believe your performance is among the top half of the session, ranging from 1%to 99%. The table below the slider determines your experimental earning, and is designed in a way that you will be best off if you indicate your true belief.

The detail is as follows. There will be 100 different option 1/2 choices. In option 1, you will win HKD 70 if your number is among the top half. In option 2, you will win HKD 70 with a probability ranging from 1% to 99%. So if you believe that with 70% probability that your number is among the top half, you should choose option 1 for the first 69 rows, and choose option 2 for row 70 to 100. Your choice in the slider will automatically determine your choices in the table. And therefore it is of your best interest to indicate your true belief. At the end, we will randomly draw one row from one of the seven guesses and pay you according to your choice.

As mentioned above, you will have to indicate your guess seventh times. First, you have to indicate your guess on how likely your number is among the top half right after the Raven's test. Next, we will give you some information each time you indicate your guess.

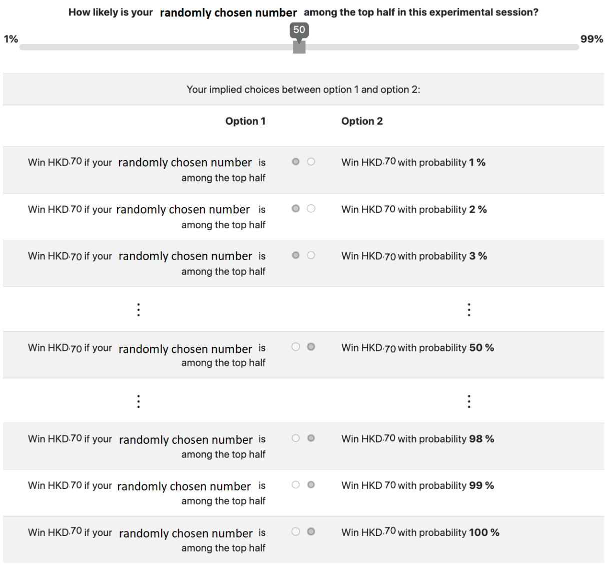

Figure 1: A slider to indicate your guess

More specifically, we will show you a thumbs-up or a thumbs-down depending on your number. Figure 1 and 2 show the details. If your number is among the top half, we will more likely show you a thumbs-up (with probability 60%); if your number is among the bottom half, we will more likely show you a thumbs-down (with probability 60%). Therefore a thumbs-up or a thumbs-down gives your a hint about your number, but they are not conclusive. It is possible that you still receive a thumbs-down even if your number is among the top half, and receive a thumbs-up even if your number is among the bottom half. You will receive six signals, and indicate six guesses along with it. For example, you see a thumbs-up, indicate a guess, then see another thumbs-up, revise your guess, then see a thumbs-down, revise your guess, etc.

generated signal	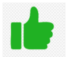	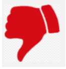
probability of the signal	60%	40%

Table 1: If you number is among the top half of the session.

generated signal	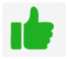	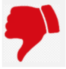
probability of the signal	40%	60%

Table 2: If you number is among the bottom half of the session.
